# A Review of the Etiology and Epidemiology of Gallbladder Cancer: What You Need to Know

**DOI:** 10.7759/cureus.28260

**Published:** 2022-08-22

**Authors:** Sattam A Halaseh, Shahed Halaseh, Raed Shakman

**Affiliations:** 1 General and Colorectal Surgery, Torbay Hospital, Torbay and South Devon NHS Foundation Trust, Torquay, GBR; 2 Surgery, Jordan University Hospital, Amman, JOR; 3 Faculty of Medicine, Torbay and South Devon NHS Foundation Trust, Torquay, GBR

**Keywords:** gallbladder polyp, gallbladder diseases and gallstones, gastrointestinal surgery, etiology and pathogenesis, cancer epidemiology, cancer of gallbladder

## Abstract

Gallbladder cancer (GBC) is the sixth most prevalent cancer of the gastrointestinal system but the most prevalent cancer of the biliary tract. This tumor is a highly fatal condition. The importance of early diagnosis cannot be overstated because GBC develops quietly with late detection. Several genetic and environmental variables have been associated with the onset of GBC. Cholelithiasis and chronic inflammation from the biliary tract and parasite infections are prime examples of environmental factors that significantly influence the development of GBC. Abnormal pancreaticobiliary duct junction and biliary cysts are examples of congenital causes. In the past decade, new imaging technologies and a more radical and aggressive surgical approach have improved patient outcomes and aided prolonged survival for GBC patients. This review article focuses on the epidemiology of GBC, its risk factors, and clinical characteristics.

## Introduction and background

Gallbladder cancer (GBC) is an uncommon tumor that primarily affects the elderly. Due to the highly malignant biology, delayed presentation, challenging anatomic location, and advanced stage upon diagnosis, the prognosis for GBC remains dismal. The treatment for locally progressed and metastatic disease is palliative chemotherapy. In contrast, the early-stage disease is possibly curable with surgical intervention and adjuvant treatment. The estimated five-year survival rate for GBC is 5%, with a median survival time of around six months [1]. In a few cases, early malignancies are discovered incidentally during cholecystectomy for cholelithiasis; in these instances, the five-year survival rate is greater than 80% [2].

In this review, we explore the epidemiology of GBC, as well as its risk factors, clinical characteristics, and diagnostic examination.

## Review

Epidemiology

**Figure 1:**
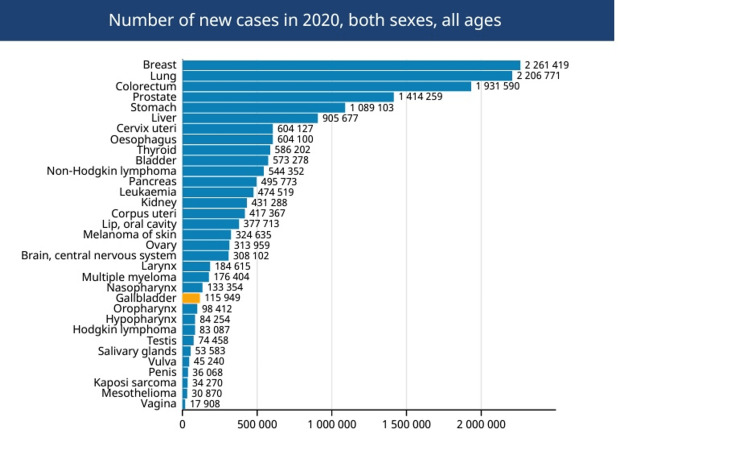
Number of new cases of gallbladder cancer globally. Available from Global Cancer Observatory: Cancer Today. Lyon, France: International Agency for Research on Cancer. https://gco.iarc.fr/today.

**Figure 2:**
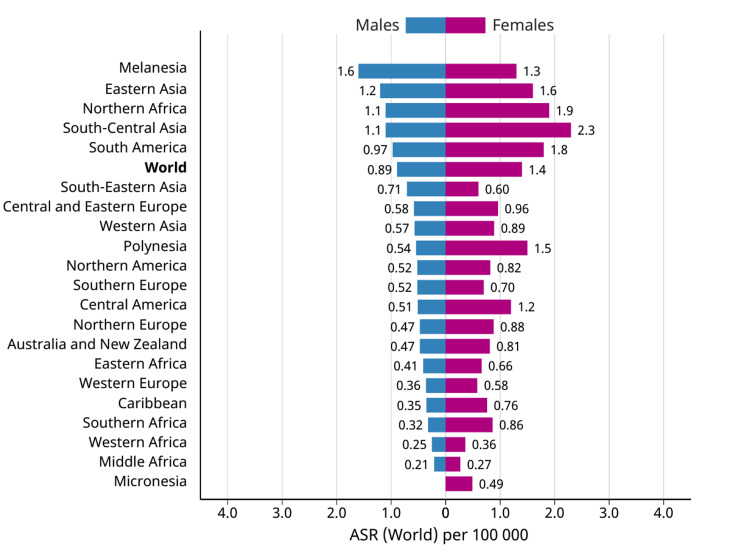
Gallbladder cancer incidence according to gender. Available from Global Cancer Observatory: Cancer Today. Lyon, France: International Agency for Research on Cancer. https://gco.iarc.fr/today.

Carcinoma of the gallbladder is the most prevalent cancerous tumor of the biliary system and the sixth most widespread cancer of the gastrointestinal system [3]. There is significant regional heterogeneity in the incidence of GBC globally, and this variability coincides with the frequency of cholelithiasis. Countries in South America, notably Chile, Bolivia, and Ecuador, as well as some regions in northern India, Pakistan, Japan, Korea, and Poland, are afflicted by significantly higher than average GBC rates [4,5]. High rates of gallstones or *Salmonella *infection, which are known to increase the likelihood of GBC, are shared by all of these groups [6]. According to estimates from Globacon, in 2020, there were around 116,000 new cases of GBC (Figure 1) and approximately 84,700 fatalities [7]. Approximately 4,000 new cases are identified each year in the United States, making North America a low-incidence region. The highest rates occur in the seventh decade of life, two to six times more prevalent in women than in men. Figure 2 describes the GBC incidence among males and females worldwide. Between 0.3% and 3.0% of individuals undergoing cholecystectomy for gallstone disease are discovered to have GBC incidentally [2]. The frequency of GBC also varies greatly by race or ethnicity; for instance, aboriginal peoples in the United States, Mexico, and Chile have a far higher risk of developing the disease. In the United States, the prevalence of GBC is roughly 1.5 cases per 100,000 persons [8].

The frequency of GBC among native American females with gallstones is around 7.1 per 100,000. The incidence of GBC among native Chilean women is 27.3 per 100,000 persons. Asian individuals, namely those of Korean heritage, are also at an elevated risk for developing GBC [2].

Etiology

Several genetic and environmental variables have been associated with the onset of GBC. The development of GBC has been linked to chronic gallbladder infection and/or environmental exposure to certain toxins, heavy metals, and other dietary variables. Existing evidence suggests that the significant relationship of GBC with the female gender and particular geographical locations mainly in developing nations is mediated by numerous female hormones, cholesterol cycling, and *Salmonella *infections [9,10]. Other well-known risk factors for GBC, such as porcelain gallbladder, Mirizzi’s syndrome, and pancreatic bile reflux, have also played a significant role in predisposing to this condition [9]. The presence of a family background of gallstones, smoking, chemical exposure, living in the Gangetic belt, and elevated levels of secondary bile acids, as well as high consumption of fried meals which include recycled oil, enhances the risk for GBC [11].

Gallstones

Up to 85% of individuals diagnosed with GBC have gallstones, making cholelithiasis the most significant risk factor for the development of GBC [12]. However, 3% of individuals with gallstones are diagnosed with GBC, and the 20-year risk of acquiring cancer remains modest; 0.5% for the general population, and up to 1.5% for high-risk populations [13].

Gallbladder stones larger than 3 cm are linked to a 10-fold greater chance of developing cancer [14]. The likelihood of getting GBC is greater in people with symptomatic gallstones than in those with asymptomatic gallstones, and cholesterol stones are the most prevalent cause of symptomatic gallstones [15].

Porcelain Gallbladder

An unusual sign of chronic cholecystitis, the porcelain gallbladder is marked by intramural calcification of the gallbladder wall. It is linked to cholelithiasis in greater than 95% of instances. As with other disorders associated with gallstones, these individuals are at a heightened risk for GBC. In individuals with porcelain gallbladders, the estimated prevalence of GBC is around 10%, with more recent research indicating a prevalence of 2-3%. Individuals with localized mucosal calcification or partial mural calcification may be at higher risk [16].

Gallbladder Polyps

Gallbladder polyps are benign growths that develop on the mucosal lining of the gallbladder and are often discovered during or after a routine ultrasound examination or a cholecystectomy. They are categorized as benign or malignant, with benign lesions further divided into non-neoplastic and neoplastic subtypes (Table 1) [17].

**Table 1:** Relative frequency of the different pathologic types of gallbladder polyps. Weedon D. Benign mucosal polyps. In Pathology of the Gallbladder. New York: Mason (1984): 195-9.

Type	Frequency
Benign polyps	
Cholesterol polyps	60%
Adenomyomas	25%
Inflammatory polyps	10%
Adenomas	4%
Malignant polyps	
Adenocarcinoma	80%
Miscellaneous:	20%
Mucinous cystadenomas	
Squamous cell carcinoma	
Adenoacanthomas	

Adenoma, a glandular growth formed of cells mimicking biliary system epithelium, is the most prevalent benign neoplastic lesion. Polypoid gallbladder lesions, which are found in up to 5% of individuals, are also associated with an elevated risk of cancer. This is especially true for polyps larger than 10 mm, which entail a 25% risk of cancer [18]. Similarly suspicious for malignancies are solitary or sessile polyps or those exhibiting fast development on serial imaging, especially if gallstones are present or the patient is older than 50. When such results are detected, even asymptomatic patients ought to have their gallbladders excised. Non-removed polyps should be monitored with serial imaging.

Chronic Infection

In endemic regions with *Salmonella*, up to 4% of acutely infected patients become chronic asymptomatic *Salmonella typhi *carriers. Multiple studies and a meta-analysis have revealed a link between both chronic *S. typhi* carriers and an increased risk of GBC [19]. Gallstones are believed to be a possible source of continued infection because chronic carriage is more common in individuals with cholelithiasis. Furthermore, *Helicobacter *colonization of the biliary epithelium, notably *Helicobacter bilis*, has been linked to the development of gallbladder disease, including GBC, as shown by the identification of *Helicobacter*-derived toxins and outer membrane proteins utilizing sensitive molecular and immunohistochemical methods [20]. Chronic inflammation related to primary sclerosing cholangitis has been linked to an increased incidence of gallbladder mass lesions [21]. Therefore, yearly gallbladder ultrasound screening is indicated for these individuals.

Congenital Abnormalities

Biliary cysts are dilatations of the bile ducts that may arise singularly or in multiple sets. Originally referred to as choledochal cysts comprising the extrahepatic bile duct, the clinical categorization has been updated to also encompass intrahepatic cysts [22]. Biliary cysts may be acquired or congenital and are linked with several anatomic abnormalities. In roughly 70% of individuals with biliary cysts, an abnormal pancreaticobiliary duct junction is present [23]. Similar to abnormal pancreaticobiliary duct junction, biliary cysts are more prevalent in Asian cultures [23]. The presence of biliary cysts is linked with an increased likelihood of developing cancer, including cholangiocarcinoma. The prevalence of cancer varies depending on age at initial presentation, ranging from less than 0.7% in individuals aged less than 10 to over 50% in older patients [24]. Another important risk factor is the presence of anomalous pancreaticobiliary duct junctions. It is an uncommon anatomical anomaly in which the pancreatic duct empties into the common bile duct, resulting in a lengthy, typically over 2 cm-long common channel. This disorder may be the result of embryological ducts failing to move completely into the duodenum. This disease is especially widespread among Asians, particularly Japanese [25].

Because the ductal junction is located away from the sphincter of Oddi, the lengthy common channel may increase the likelihood of pancreatic juice refluxing into the biliary tree [26]. High amounts of amylase in bile, intraductal stimulation of proteolytic enzymes, changes in bile content, assumed biliary epithelium injury, irritation, ductal dilatation, and cyst development are the results [27]. The presence of an anomalous pancreaticobiliary duct junction has been linked to an increased risk of biliary and pancreatic cancer, even in the absence of a biliary cyst or ductal dilatation [28,29]. Patients with an abnormal pancreaticobiliary duct junction and no bile duct cysts are most frequently diagnosed with GBC. Therefore, precautionary cholecystectomy is advised for affected patients.

Carcinogenic Exposure

Mounting evidence suggests that carcinogen exposure may potentially play a role in the genesis of GBC. Employees in the oil, paper, chemical, shoe, and textile sectors and miners exposed to radon are at a greater risk of developing GBC [30,31]. A higher risk has also been observed in cigarette smokers [32] and perhaps in individuals with high levels of contact with aflatoxin, a mycotoxin that widely contaminates maize, soybeans, and peanuts and has been linked to an elevated risk of hepatocellular carcinoma [33].

Medications

Methyldopa, oral contraceptives, and menopausal hormone treatment, as well as isoniazid, are a few examples of medications that have been linked to the development of biliary cancer [34,35].

Body Mass Index and Diabetes

People who are obese have an increased likelihood of developing GBC [36]. People who have diabetes mellitus are at a higher risk of developing GBC than those who do not [37].

Pathogenesis of gallbladder cancer

In individuals with cholelithiasis and an abnormal pancreaticobiliary duct junction, variations in demographic trends, clinical manifestations, and gender distribution imply the existence of two major paths for the development of GBC. Cholelithiasis and subsequent cholecystitis appear to be the driving factor in most countries around the world where GBC is significantly related to gallstone disease, gender prejudice toward women, and age over 65 [6]. A second mechanism includes anomalous pancreaticobiliary duct junction, which is linked with many GBC cases in Japan [38]. Cancers linked with this syndrome develop at a younger age, exhibit less female gender disparity, and have a lower cholelithiasis incidence rate. In addition, histologic and molecular distinctions exist between GBCs accompanied by anomalous pancreaticobiliary duct junction and those related to gallstones, giving additional evidence that two separate pathogenetic processes are implicated [39]. Japanese GBCs with an abnormal pancreaticobiliary duct junction are distinguished by KRAS mutations and a somewhat delayed start of p53 alterations [40]. In contrast, KRAS variants are uncommon, at least in Chilean individuals with cholelithiasis and recurrent cholecystitis, whereas p53 mutations develop early throughout multiphase pathogenesis [41].

The majority of epithelial malignancies are preceded by a sequence of histopathological and molecular alterations that develop over many years or decades. Similar to other adenocarcinomas of the gastrointestinal tract, adenocarcinomas of the gallbladder develop from dysplasia to carcinoma in situ to invasive malignancy. These sequential alterations are characterized by fewer well-understood molecular modifications than colorectal cancer [6]. In standard cholecystectomy specimens, the mucosa close to more than 90% of GBCs demonstrates dysplastic alterations [42], which are rather common. It appears that the overall process takes around 15 years. Symptomatic cholecystitis is uncommon before the age of 40; the median age for discovering dysplasia is 45 years, and the average age for detecting carcinoma in situ is 55 years [43]. Squamous metaplasia is an uncommon premalignant lesion associated with squamous cell invasive GBC.

In contrast, epithelial hyperplasia with a papillary or villous morphology is observed in up to 60% of cases in both adults and children with an abnormal pancreaticobiliary duct junction [38]. This is assumed to indicate a premalignant histological alteration in the gallbladder mucosa. The progression from hyperplasia to dysplasia is comparable to the typical type of GBC.

Clinical manifestations and diagnosis

GBC may be detected preoperatively, intraoperatively during surgical investigation for abdominal symptoms owing to another disease state, or postoperatively upon evaluation of the gallbladder specimen, which is commonly removed during cholecystectomy because of symptomatic cholelithiasis. The vast majority of patients with early disease are asymptomatic or have vague symptoms that mirror or are caused by cholelithiasis or cholecystitis. To begin with, pain is the most commonly reported ailment among those suffering from symptoms mainly in the right upper quadrant, followed by loss of appetite and nausea, and vomiting. The features of late GBC frequently differ from those of typical biliary colic and are far more indicative of malignancy such as malaise and weight loss. Patients who report a symptom combination indicative of acute cholecystitis are more likely to have an earlier illness stage and a better long-term prognosis [14]. Patients with GBC may also have obstructive jaundice, either as a consequence of direct penetration of the biliary tree or metastatic disease to the hepatoduodenal ligament. This condition should be strongly suspected if an impacted gallbladder stone is found to be compressing the common hepatic duct. The growth of tumors further into porta hepatis may potentially cause blockage of the duodenum [44]. A jaundiced patient’s physical assessment may disclose a palpable gallbladder. Courvoisier’s sign was first postulated as an indication of pancreatic or gallbladder cancer rather than cholelithiasis [45]. Due to the exceptions to this rule like chronic pancreatitis, parasite biliary blockage, congenital choledochal cyst, and common hepatic duct blockage proximal to the origin of the cystic duct [45], the diagnostic value of this characteristic is restricted.

In general, laboratory tests are nondiagnostic; however, an increased alkaline phosphatase or serum bilirubin may indicate bile duct blockage. Serum biomarkers such as carcinoembryonic antigen (CEA) and carbohydrate antigen 19-9 (CA 19-9) are frequently raised, but they lack the specificity and sensitivity to be diagnostically useful [46]. Imaging can identify cancerous growths. Transabdominal ultrasonography is a valuable first diagnostic method for individuals with upper abdominal discomfort and jaundice [47]. Currently, endoscopic ultrasonography (EUS) is the gold standard for the staging of GBC. EUS also provides a sample employing fine-needle aspiration [48]. Preoperative computed tomography (CT) identifies any expansion to the lymphatic system, hepatic lesions, or distant metastases with a high degree of accuracy of almost 93%, effectively determining the resectability of the gallbladder [49]. In general, conventional magnetic resonance imaging (MRI) is less beneficial. Magnetic resonance (MR) cholangiography and MR angiography identify bile duct or vascular invasion with about 100% sensitivity and specificity.

Positron emission tomography (PET) scans are important for distinguishing malignant from benign pathology, preoperative staging, and postoperative disease detection [50].

Histology of gallbladder cancer

The categorization of gallbladder primary neoplasms according to their histologic characteristics is presented in Table 2 [51]. Other histologic forms, such as squamous cell carcinoma, small-cell neuroendocrine tumors, lymphoma, and sarcoma, are sometimes seen [52]. Compared to adenocarcinomas, squamous cell malignancies are often of a higher grade, detected at an advanced stage, and have a worse survival rate, even when full resection is taken into account [53].

**Table 2:** Histological type of gallbladder cancer.

Gallbladder cancer histological type
Adenocarcinoma
Papillary
Mucinous
Adenosquamous
Squamous
Oat cell

As they develop, adenocarcinomas begin as mucosal lesions that invade the gallbladder wall. Papillary, nodular, and tubular are the subtypes of histology for gallbladder adenocarcinomas. Less than 10% of gallbladder cancers are papillary but are linked with an infinitely better prognosis because they are typically identified when still limited to the gallbladder [54]. The absence of a distinct muscular layer allows for the early invasion of vascular, lymphatic, and neurological tissue. As they develop, tumors typically spread beyond the gallbladder and invade neighboring organs, especially the liver. The gallbladder metastasizes via lymph vessels, venous drainage, or direct invasion of the liver parenchyma. Lymphatic drainage from the gallbladder drains initially to the cystic duct node, followed by the pericholedochal and hilar nodes, and lastly to the peripancreatic, duodenal, periportal, celiac, and superior mesenteric artery nodes. The veins of the gallbladder drain directly into the surrounding liver often segments IVb and V, where tumor infiltration is prevalent [55]. Unfortunately, only 10-25% of GBC cases are detected when they are still confined to the gallbladder. At the time of diagnosis, the preponderance will already possess nodal involvement, expansion into the neighboring liver, or distant metastases [56]. The most prevalent sites of disease metastasis are the peritoneum and liver. Infrequently, distant metastases impact the lungs and pleura [55].

Staging

Several staging schemes have been utilized for GBC. The Tumor, Node, Metastasis (TNM) staging method developed by the American Joint Committee on Cancer (AJCC) and the Union for International Cancer Control (UICC) now serves as the recommended grading technique [57].

Management

The management choices are determined by the stage of the disease. Those who are eligible for cholecystectomy achieve the greatest outcomes. According to AJCC standards, stage I illness is characterized by tumor invasion of the lamina propria or muscle layer. Perforation of the serosa and/or invasion of the surrounding organs or tissues define stage II. Invasion of lymph nodes by a T1 to T3 tumor is automatically categorized as stage II. Both stages I and II may be resectable for curative purposes. Because of vascular invasion or the involvement of many nearby organs, stage III cancers are typically incurable at the local level. Stage IV reflects incurability due to the presence of metastatic disease [58].

Neoadjuvant treatment is generally not an option because of advanced illness upon diagnosis and is not regarded as the quality of practice in resectable cancer situations. Consideration should be given to early-stage clinical studies [58]. If there are no contraindications, surgery is the sole curative therapy for people with stage II or less.

**Table 3:** Contraindications for resection.

Liver metastases
Peritoneal metastases
Malignant ascites
Distant involvement of lymph nodes (paracaval, para-aortic, superior mesenteric artery)
Occlusion of major blood vessels

Surgical excision of GBC consists of cholecystectomy with marginal hepatectomy and local lymphadenectomy or common bile duct excision. For GBC identified incidentally on cholecystectomy pathological specimens of T2 or higher stages, further investigation and re-resection are recommended. Postoperative chemotherapy should be administered within eight to twelve weeks and needs baseline laboratory testing and imaging to restage the illness before treatment begins [59].

Patients with an excised pathology specimen report of T2 or above, node-positive, and margin-positive should have adjuvant treatment, ideally for six months of adjuvant chemotherapy (ACT) or four months with simultaneous adjuvant chemoradiation (ACRT). If clinically warranted, NCCN surveillance recommendations include scanning every six months for two years, then yearly for up to five years [60].

Advanced disease and metastatic gallbladder tumors that cannot be resected are suitable for palliative care/chemotherapy. Locally advanced cancers may be treated with external-beam radiation treatment, which often comprises a radiosensitizer such as 5-fluorouracil; however, this method achieves tumor control seldom [61].

The cumulative five-year survival rate for all individuals with GBC is 20%, but the five-year survival rate for patients with localized disease susceptible to surgical resection is 65% [62].

The dismal results reported in the majority of GBC studies reflect the vague symptoms and terminal stage of the disease upon presentation. In several large series, five-year survival rates range from 5-12% [63]. The prognosis is relatively dismal for all illness stages after T1N0. Unfortunately, less than 10% of symptomatic individuals have T1 tumors [64].

## Conclusions

GBC is the sixth most prevalent cancer of the gastrointestinal system but is the most prevalent tumor of the biliary tract, with an incidence that varies greatly around the globe. Most often, the histopathological type is adenocarcinoma. Cholelithiasis-caused chronic inflammation is the most significant risk factor.

Although GBC may be diagnosed after cholecystectomy, preoperative imaging using transabdominal and endoscopic ultrasound and multi-sliced CT represents a significant development. Surgery is the only possible treatment option. Unfortunately, the typical late presentation indicates an older age with the possibility of nodal involvement, resulting in recurrence despite attempts at resection. In the evaluation of these patients, the entire medical team, including all doctors and junior doctors, must be informed of the risk of this adverse event.
